# Erosive Arthropathy in systemic sclerosis

**DOI:** 10.1186/1471-2458-7-260

**Published:** 2007-09-22

**Authors:** Fadoua Allali, Latifa Tahiri, Adil Senjari, Redouane Abouqal, Najia Hajjaj-Hassouni

**Affiliations:** 1 Rheumatology department, El Ayachi University-Hospital, Sale, Morocco; 2 Laboratory of Biostatistic, Clinical Research and Epidemiology. LBRCE. Faculty of Medecine and pharmacy, Rabat, Morocco

## Abstract

**Background:**

To determine radiological features of arthropathy in systemic sclerosis patients with polyarthritis.

**Results:**

Forty one women and 5 men were included in this study. The mean age was 41 + 14,2 years, the mean duration of disease was 10,5+ 6,5 years. Thirty seven patients (80%) had radiological abnormalities including joint space narrowing (37%) and erosions (43%). At presentation, the prevalence of radiological foot abnormalities was lower than that of hands (26% vs 79%, p < 0,001). There was no significant difference between patients with (n = 24) and without erosive arthropathy (Joint space narrowing and/or erosion) (n = 22) in terms of cutaneous subtype, organ involvement, calcinosis presence of rheumatoid factor, ANA, Anti-topoisomerase antibodies.

**Conclusion:**

This study showed an high frequency of erosive arthropathy in our morroccan SSc patients with clinical synovitis.

## Background

Systemic sclerosis (SSc) is a connective tissue disease characterized by vascular involvement and fibrotic changes in the skin and internal organs. Joint symptoms have been noted in 12 to 66% of patients at the time of diagnosis and in 24 to 97% of patients at some time during the course of their illness [1]. Many radiological changes in the joints of SSc patients have been identified but erosive arthropathy is considered uncommon. The aim of this study was to assess radiological features of SSc patients with polyarthritis.

## Methods

Clinical records of 60 SSc patients fulfilled the American college of rheumatology classification criteria [2], admitted to rheumatology department between 1981 and 2007 were reviewed. We included only patients with 4 or more clinical synovitis (joint swelling and/or increase in warmth). In all patients, we assessed at presentation cutaneous SSc subtype as defined by Leroy et al [3] (limited disease when skin involvement was distal to the elbow/knees, diffuse disease when the trunk was involved), disease duration calculated from the first symptom attributable to SSc and visceral involvement defined by the following means: heart (clinical examination, electrocardiogram and if necessary echocardiography), lung (dyspnea, pulmonoray function test, chest radiograph and if necessary computed tomography), kidney (urine analysis, BUN, and serum creatinine level), gastrointestinal tract (dysphagia, oesophageal manometry). The serological status of the patients for rheumatoid factor was performed by waaler rose test and latex agglutination test (cut-off level 1/40), antinuclear antibodies (ANA) was determined by indirect immunofluorescent technique on HEp-2 cells (cut-off level : 1/40), and anti DNA topoisomerase I antibodies (Anti-Scl 70) by enzyme-linked immunosorbent assay (cut-off level, 20 EU/ml). Radiographs of hands and feet at presentation, were read by 2 independent observers. The interobserver coefficient of variability was 0,1. Three radiological patterns of abnormalities of joints (erosion: interruption of the cortical surface; space narrowing: focal or diffuse joint narrowing), bone (radiological demineralisation: juxta-articular or generalised osteoporosis; bone resorption), and soft tissue (calcifications) were assessed.

Statistical analysis was carried out with the student's test for continuous data and chi square test for categorical data. Multiple logistic regression was used to analyse predictor variables as age, disease duration and erythrocyte sedimentation rate (ESR). Differences were considered significant when p < 0,05

## Results

At presentation, 46 out of 60 SSc patients had 4 or more synovitis and were included in the study. Most of SSc patients were women (female/male ratio 8,2) with a mean age of 41 ± 14,2 (SD) years (median, 38.5 years) and a disease duration of 10,5 ± 6,5 years (median, 10 years). There were 16 cases (35%) with limited scleroderma and 30 cases (65%) with diffuse scleroderma. The prevalence of the different organs and systems involved were as follows: Lung: 57%, Gastro-intestinal tract: 37%, heart: 17%, kidney: 4%. Inactive digital ulcers were seen in 15% of cases (see table 1).

**Table 1:** Characteristics of systemic sclerosis (SSc) patients

**Characteristics of patients**	**SSc patients**
**Sex: female/male**	41/5
**Mean age (years)**	41 ± 14,2
**Mean duration of disease (years)**	10,5 ± 6,5
**SSc subtype**	
Diffuse systemic sclerosis	30 (65%)
Limited systemic sclerosis	16 (35%)
**Peripheral vascular (inactive Digital ulcers)**	7 (15%)
**Organ involvement**	
Lung involvment	25 (57%)
Gastro-intestinal involvement	17 (37%)
Heart involvement	8 (17%)
Kidney involvement	2(4,3%)
**Positive rheumatoid factor**	12(31%)
**Positive antinuclear antibodies**	24 (62%)
**Antitopoisomerase I**	15 (33%)

Values are presented as mean ± SD and n(%)

SSc: systemic sclerosis

Twelve patients had overlap syndromes: rheumatoid arthritis, 2 cases; systemic lupus erythematosus, 3 cases, polymyositis, 3 cases, sjögren syndrome, 4 cases.

For the serological status of the patients, SSc patients were ANA positive in 62%, antitopoisomerase I antibodies were positive in 33%. Rheumatoid factor was positive in 31%. when we compared the age (43,3 ± 14,1 vs 39,8 ± 14,3; p = 0,4) and disease duration (11,2 ± 7,3 versus 9,1 ± 4,6; p = 0,2) of SSc patients divided in limited and diffuse subset no difference emerged.

**Figure 1:**
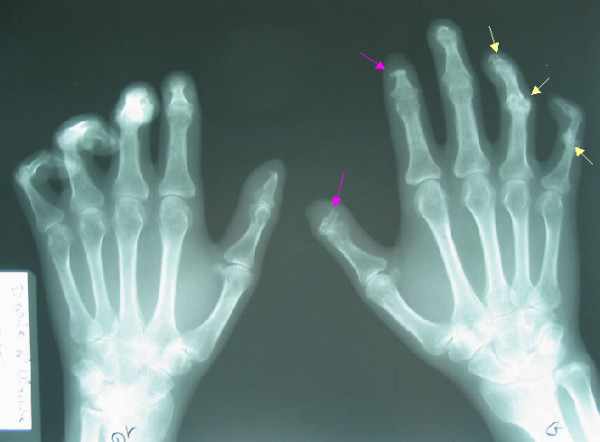
X-ray of hands showing. - Erosion of the fourth and the fifth proximal inter phalangeal joint on the left hand. - Terminal phalangeal tuft resorption (arrow).

**Figure 2:**
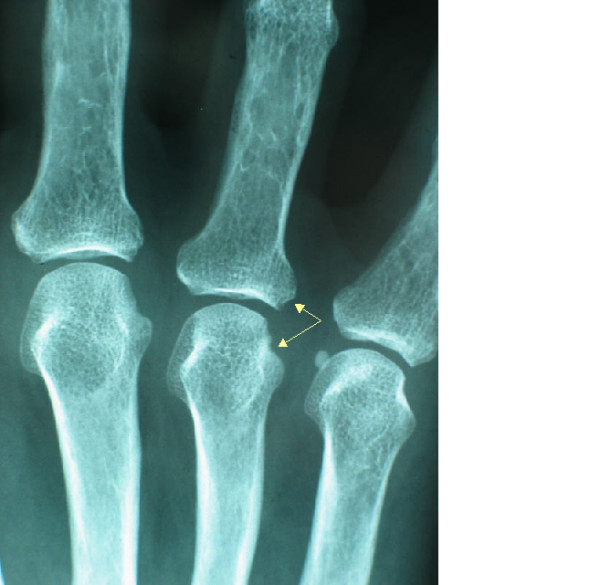
X-ray of hand showing: a small and discrete lesion in the fourth metacarpo-phalangeal joint similar to early rheumatoïd arthritis.

**Figure 3:**
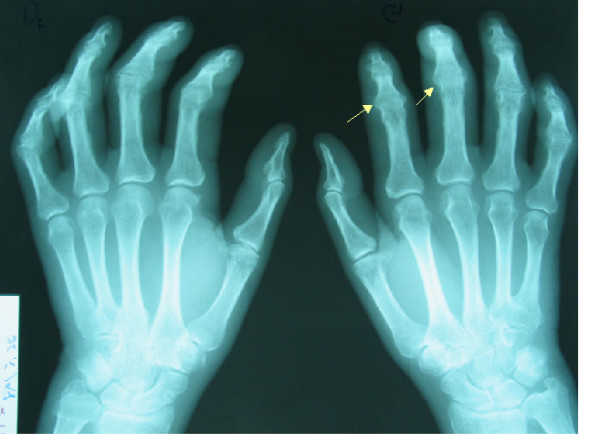
X-ray of hands showing a joint space narrowing in the proximal inter phalangeal joint similar to erosive osteoarthritis.

**Figure 4:**
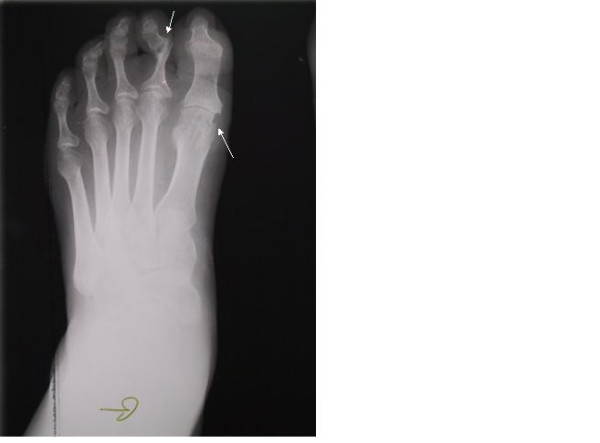
X-ray of a left foot. Erosive lesion in the first metatarso-phalangeal joint, and subluxation and erosion of the second proximal inter phalangeal joint similar to psoriatic arthritis.

Juxtaarticular demineralization was detected in 17 patients (37%), distal phalange resorption in 8 patients (18%) and extra articular calcifications in 5 patients (12%). Radiological changes seen in our SSc patients are summarized in the table 2. The more prevalent radiological patterns of SSc patients are illustrated in figures 1, 2, 3, 4.

**Table 2:** Radiological abnormalities of the hands, wrists and feet in systemic sclerosis (SSc) patients

Radiological abnormalities	Systemic patients n = 46
**Radiological abnormalities in the hand**	
Bone demineralization	33%
Joint space narrowing	15(33%)
MCP	3
PIP	11
DIP	2
Radiocarpal	7
Joint erosions	14(31%)
MCP	4
PIP	10
DIP	1
Radiocarpal	7
Ulnar styloid erosions	1(2%)
**Radiological abnormalities in the foot**	
Bone demineralization	5%
Joint space narrowing	2 (5%)
MTP	1
Ankle	1
Joint erosion	5 (12%)
MTP	3
Talo-navicular joint	2
**Terminal phalangeal tuft resorption**	8 (18%)
**Calcinosis**	5(12%)

Values are n (%)

DIP: distal interphalangeal joint, MCP: metacarpophalangeal joint, PIP: proximal interphalangeal joint. MTP: metatarsophalangeal joint

At presentation, the prevalence of radiological foot abnormalities was lower than that of hands (26% vs 79%, p < 0,001), whereas no significant difference was detected in the prevalence of joint space narrowing (5% of the feet vs 33% of the hands; p = 0.3), erosion (12% of the feet versus 31% of the hand; p = 0.18) and bone demineralization (5% of the feet versus 33% of the hands; p = 0,29).

We found no significant difference between patients with (n = 24) and without erosive arthropathy (Joint space narrowing and/or erosion) (n = 22) in terms of cutaneous subtype, organ involvement, calcinosis, presence of rheumatoid factor, ANA, Anti-topoisomerase antibodies (see table 3). In multiple logistic regression, age of patients > 38 years, disease duration longer than 10 years and ESR levels > 30 mm/h at presentation were risk factors but not significant association with erosions emerged (see table 4)

**Table 3:** Comparison between clinical and serological features of SSc patients with and without joint erosions

	Erosive arthropathy N = 24 (%)	Non erosive arthropathy N = 22 (%)	P*
**SSc subtype**			
Diffuse SSc	77,5	51,8	0,2
Limited SSc	22,5	48,2	0,3
**Digital ulcers**	13	18	0,5
**Calcinosis**	4	18	0,1
**Organ involvement**			
Lung disease	58	50	0,5
Kidney involvement	4	4	1
Heart involvement	21	14	0,5
Gastro-intestinal involvement	32	42	0,9
**Associated connectivitis**	21	30	0,4
RA	8	0	0,1
SLE	4	9	0,6
Sjögren's Syndrome	0	18	0,08
Polymyositis	8	5	0,4
**Positive rheumatoid factor**	33	18	0,2
**Positive antinuclear antibodies**	46	60	0,9
**Anti topoisomerase I antibody**	4	5	0,9

Values are %

*Comparison between two groups carried out by chi-square test,

RA: rheumatoid arthritis, SLE: systemic lupus erythematosus

**Table 4:** Correlation between some clinical patients characteristics and erosive changes in systemic sclerosis patients

**Variable**	**Odds Ratio (OR)**	**95% Confidence interval**	P*
Age > 38 years	1,15	0,33–3,9	0,8
Disease duration > 10 years	2,1	0,61–7,3	0,2
ESR > 30 mm	3	0,8–11,8	0,1

* Multiple logistic regression

ESR: erythrocyte sedimentation rate

## Discussion

This study analyzed SSc patients with polyarthritis that has been observed in 46 out of 60 cases. This result is in agreement with Misra et al [4] who had documented clinical synovitis and joint inflammation with imaging techniques in 88% and 91%, respectively, of their SSc patients with a current or past history of articular symptoms. Erosive arthropathy was seen in 24/46 cases, is in agreement with prevalence of other reports [1,5,6]. The erosions were frequently atypical of rheumatoid arthritis, tended to be small and discrete, reflecting the relatively non invasive nature of the synovium in SSc. Histological studies have shown mild proliferation and fibrosis without pannus formation [7]. The cause of erosions in SSc remains unclear and has been hypotesized a role of traction of tendons on demineralised bone or ischemic bone resorption [8,9]. Rabinowitz et al [10] reviewed the hand and wrist x-rays of 24 patients with scleroderma. All had mild arthralgia or arthritis, and bone erosions were seen in 13 patients. The x-rays of 7 patients showed many features in common with rheumatoid arthritis.

A marked erosive lesions resembling rheumatoid arthritis may be observed in SSc patients [10]. In our study, the diagnosis of scleroderma in the two cases who had changes resembling those seen in rheumatoid arthritis was not in doubt. They had a proximal scleroderma changes considered to be the major manifestation of this disorder. An overlap syndrome can not be excluded in those cases. One third of our patients had positive test for rheumatoid factor. This may occur in 30 % of patients with scleroderma. Clark et al [11] found a correlation between inflammatory synovial biopsy appearances and the presence of rheumatoid factor in the serum or synovial fluid. Kellgren and Ball [12] believed that high titres of rheumatoid factor tended to occur in patients with proeminent articular features. However this was not confirmed by Rodnan [13] in his study of 29 patients. Recently Ingegnoli et al [14] found in 75 SSc patients a statistically significant association between the positivity of anti cyclic citrullinated peptide antibodies (anti CCP) and the presence of arthritis and marginal erosions. This may help to define the diagnosis of overlap syndrome SSc/RA and to facilitate diagnosis and appropriate treatment. Two thirds of our patients had ANA positivity. Prevalence of ANA in our series is lower than that reported in European studies [6,15], whereas is in agreement with a recent a Moroccan study [16]. We hypothesized that this may be due to etnic variability.

In our study, the more frequent changes were joint space narrowing in PIP, that may be due to deformities of the fingers caused by soft tissue contractures. Instead the joint space narrowing of DIP joints were uncommon in contrast with Avouac et al [17] who described a higher frequency of changes. Many of those cases were associated with erosive changes and a radiological picture compatible with erosive osteoarthritis. However, the DIP lesions appear to be both deeper and more destructive than the usual picture of erosive osteoarthritis. Selective erosions of the first carpo-metacarpal joint has been described in scleroderma [9,18]. In this study, the PIP joint and radio-carpal joint were the most common site for erosions. An interesting finding in our patients was the difference between Feet and hand radiologic abnormalities that in agreement with *La Montagna *findings in his longitudinal study of 100 patients [6] confirms that the foot involvement is less frequent. Hand changes is generally observed at disease onset and remained nearly unchanged over time, foot changes began later and worsened with ongoing disease [6]. This contrast with rheumatoid arthritis patients in whom radiologic features occur earlier in the feet than in the hands, although the rate of damage progress similarly.

In Previous reported data, destructive arthropathy was associated to high C reactive proteine concentration (> 10 mg/l) suggesting that inflammatory articular involvement may partly account for the unusual inflammatory syndrome observed in SSc patients [17]. In our study, no relation was found between the occurrence of destructive arthropathy and age, organ involvement, calcinosis, associated connectivitis, presence of rheumatoid factor and antinuclear antibodies. In the multiple logistic regression analysis, higher ESR age > 38 years or disease duration > 10 years, were risk factors for occurrence of erosions but a not significant relationship was found, indicating that probably other factors may be considered.

Our study has many limitations; it was a retrospective study and analyzed a small number of patients. Large studies including patients with scleroderma and erosive arthropathy are required to confirm our results.

## Conclusion

We documented a high frequency of clinical synovitis in our SSc patients, and a relatively low prevalence of ANA in Moroccan SSc patients. Other studies on SSc patients with arthropathy are needed to confirm our results.

## Competing interests

The author(s) declare that they have no competing interests.

## Authors' contributions

FA and NH-H conceived the study and supervised its design, execution, and analysis and participated in the drafting and critical review of the manuscript. RA participated in the concept and design of the study, did data management and statistical analyses and participated in the drafting and critical review of the manuscript. LT and AS enrolled patients, participated in data acquisition and critical revision of the manuscript. FA wrote the paper, all authors read and approved the final manuscript.

## Pre-publication history

The pre-publication history for this paper can be accessed here:


